# The status of intimate partner violence against pregnant women in contemporary China: a scoping review

**DOI:** 10.4069/whn.2024.03.16

**Published:** 2024-03-29

**Authors:** Xue Mei Fan, Hae Won Kim

**Affiliations:** 1 College of Nursing, Seoul National University, Seoul, Korea; 2 College of Nursing, The Research Institute of Nursing Science, Center for Human-Caring Nurse Leaders for the Future by Brain Korea 21 (BK 21) Four Project, Seoul National University, Seoul, Korea

**Keywords:** China, Domestic violence, Hong Kong, Intimate partner violence, Pregnancy

## Abstract

**Purpose:**

This review explored the status of publications on intimate partner violence (IPV) against pregnant women in contemporary China.

**Methods:**

The PubMed, Cochrane Library, Embase, CINAHL, and PsycINFO databases were searched using the terms “IPV,” “pregnant woman,” “Chinese,” and synonyms in English, along with related keywords for Chinese publications. All literature pertaining to IPV during pregnancy, conducted in China, and published between 1987 and September 2023 was included.

**Results:**

A total of 37 articles from 30 studies were selected. The prevalence of IPV during pregnancy ranged from 2.5% to 31.3%, with psychological violence being the most common form. Frequently identified risk factors included unintended pregnancy, poor family economic conditions, male partners engaging in health risk behaviors, poor employment status of women or their partners, low education levels among women, physical or mental health issues, strained couple relationships, and in-law conflicts. IPV during pregnancy primarily led to mental health problems for the victims and could result in adverse obstetric outcomes, as well as negative effects on the temperament and development of the offspring. Victims in China demonstrated a low willingness to seek help from professionals. Furthermore, relevant research in mainland China is scarce, with a limited number of studies and non-standardized research methodologies.

**Conclusion:**

Future research should investigate IPV in pregnancy from various perspectives, identify factors unique to IPV during pregnancy, and focus on high-risk groups. Considering the conditions in China, there is a pressing need to increase public awareness of IPV and to investigate interventions aimed at addressing this issue.

## Introduction

Intimate partner violence (IPV) is a global health issue that has been defined as “physical violence, sexual violence, stalking and psychological aggression by a current or former intimate partner” [1]. It can be perpetrated by individuals of any gender against partners of any gender, but the most common pattern is for men to commit IPV against their female partners [1]. Nearly 30% of women aged 15 and above have experienced different forms of IPV in their lifetime [2]. In the United States, 24.6% of women have experienced sexual violence, 22.3% have faced severe physical violence, and 9.2% have experienced stalking from their intimate partners throughout their lifetime [1]. IPV has detrimental effects on victims’ physical and mental health and can even lead to death [3-6].

China is a country deeply influenced by patriarchy. Throughout its millennia of history, Chinese women have been oppressed, enslaved, sold, and even lynched by men. The “three obediences and four virtues” of Chinese Confucianism serve as a framework for social behavior and stand as stark evidence of their suffering [7]. Historically, Chinese women have been subjected to violence at the hands of men. It was only in contemporary China (i.e., the period from the establishment of the People’s Republic of China in 1949 to the present day) that the terms and concepts of gender equality and domestic violence began to emerge in Chinese society. In the early years of this era, Mao Zedong’s political slogan proclaiming that “women hold up half the sky” inspired women’s awareness of equality, at least to some extent. Since the 1980s, China’s reform and opening-up policy has exposed the Chinese people to the outside world, and the introduction of Western ideas, such as feminism, has further strengthened women’s self-awareness. The term “domestic violence” started to appear in public discourse, with research on the topic beginning to emerge. The first publication to mention “domestic violence” or “family violence” in China appeared in 1987 [8]. Since then, research has focused on the issue of family violence in China, gradually revealing the true extent of IPV within Chinese society. Studies indicate that IPV from men to women in Chinese society is highly prevalent. A nationally representative survey reported that approximately 34% of Chinese women had experienced physical violence in their current relationship, with 12% of these incidents resulting in some form of injury [9]. A review of studies conducted between 1987 and 2006 found that the prevalence of women experiencing IPV ranged from 3.6% to 64.8%, with a pooled average of 19.7% for any type of violence [10]. The rates for psychological, physical, and sexual violence were 42.6%, 14.2%, and 9.8%, respectively [10]. These percentages are likely underestimated due to the social norm of not airing one’s dirty laundry in public, which may cause some individuals to be reluctant to report their experiences of IPV [11].

Among victims of IPV, pregnant women represent a particularly vulnerable group. Studies in China have reported that the prevalence of IPV during pregnancy ranges from 2.5% to 11.6%, with a combined average of 7.7% [12]. This figure accounts for more than one-third of all male-to-female violence. The consequences of IPV for pregnant women are often more severe than for the general population. Violence can directly cause physical harm to both the pregnant woman and the fetus [13] and may lead to a range of obstetric complications, such as antepartum hemorrhage, preterm birth, and even fetal or maternal death [14-16]. Furthermore, IPV during the psychologically vulnerable period of pregnancy can inflict serious psychological trauma, potentially resulting in emotional issues like stress, anxiety, and depression [17]. Maternal mental health problems can subsequently lead to difficulties in parenting, including poor maternal-infant bonding, breastfeeding avoidance, child neglect and abuse, and can have a harmful impact on the long-term growth and development of children [18-21].

In countries such as China, the issue of IPV is deeply entrenched in patriarchal social traditions. Additionally, the development of relevant policies is complicated by the country’s large population and diverse demographic makeup. Nevertheless, IPV during pregnancy must be addressed urgently. However, only one review has summarized the prevalence of IPV during pregnancy in China [12]. No comprehensive investigation has been conducted to summarize the body of research on this topic. Therefore, this scoping review explored the scope and status of research on IPV during pregnancy in China by identifying and summarizing relevant literature. The review specifically aimed to summarize the general characteristics, research methods, and content of the literature on IPV against pregnant women in contemporary China.

## Methods

**Ethics statement:** This study is a literature review of previously published studies; therefore, it was exempt from Institutional Review Board approval.

### Study design

This scoping review was carried out using Arksey and O’Malley’s [22] methodological framework for scoping reviews, which includes: (1) defining research questions, (2) identifying relevant studies, (3) selecting appropriate literature, and (4) charting data and comparing findings. We adhered to the updated PRISMA (Preferred Reporting Items for Systematic Reviews and Meta-Analyses) 2020 guidelines for identifying, selecting, and synthesizing relevant studies [23]. The ecological model served as the analytical framework for examining factors related to IPV [24].

### Research question

The research question for this study was, “What is the scope and status of research on IPV during pregnancy in China?”

### Literature search

The databases PubMed, Cochrane Library, Embase, CINAHL, and PsycInfo were systematically searched. The search period spanned from 1987, marking the first appearance of the term “family violence” in Chinese publications, to September 2023. Following consultation with a librarian, three English terms—“intimate partner violence,” “pregnant woman,” and “Chinese”—along with their synonyms, were employed in the literature search. For instance, the search process in the Cochrane Library is detailed in Supplementary Material 1. To identify relevant Chinese studies, a researcher (XMF) used the Chinese words “孕妇 (pregnant women)” and “家暴 (domestic violence)” to search the China National Knowledge Infrastructure (CNKI).

### Literature selection

The inclusion criteria for this literature review were articles on IPV against pregnant women in China, published in either English or Chinese. There were no restrictions on the research method. Studies focusing on Chinese women living in other cultures were excluded, as this review is specific to IPV within China. Upon systematically searching each database, the reviewer (XMF) imported the search results into EndNote and used the software’s duplicate removal function to eliminate duplicates. Subsequently, two authors (XMF and HWK) independently screened the titles and abstracts to assess their adherence to the inclusion and exclusion criteria. For those articles that met the inclusion criteria, the authors proceeded to review the full texts to decide on their inclusion in this scoping review. The reviewer (XMF) also examined the reference lists of all included studies and related reviews to ensure comprehensive coverage of the literature that met the inclusion and exclusion criteria. In cases where articles were published in both English and Chinese, the English-language versions were selected. When articles were published multiple times, only the version with the most pertinent information was chosen. Any disagreements between the two reviewers were resolved through discussion.

### Recording data and contrasting results

After carefully reading the full text, a reviewer (XMF) extracted the key information from the included literature, and another reviewer (HWK) checked the accuracy of the information. The key information included the general characteristics of the study, research methods, analysis and research tools (author, publication year, publication language, research purpose, study design, instrument used to measure IPV, participants, characteristics of participants, and research setting), IPV prevalence, IPV-related variables, and key outcomes of the study (IPV-related adverse health outcomes and other results). The general characteristics of the selected studies, IPV prevalence, IPV-related variables, and the key outcomes were examined.

## Results

**Figure 1.**
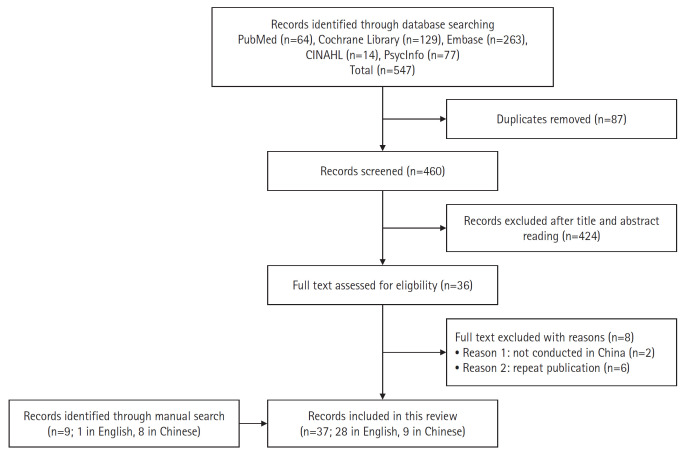
Selection process for article inclusion.

From an initial pool of 547 search results, 87 articles were identified as duplicates and an additional 424 articles were excluded for not meeting the inclusion criteria. Upon full-text review of the remaining 36 articles, two were excluded because the research was not conducted in China, and six were excluded as repeat publications. Thus, 28 articles that met the inclusion criteria were selected. Nine additional articles were identified by manually searching the CNKI, reviewing the references of the aforementioned 28 articles, and examining the articles cited in other relevant reviews [8,10,12]. Thus, a total of 37 articles [25-61] were included in this review. These 37 articles (26 in English and nine in Chinese) represent a total of 30 distinct studies because some studies were reported in multiple articles. The article selection process is depicted in Figure 1.

### General characteristics of the studies

The general characteristics of the included literature are summarized in Table 1. Approximately half of the studies were from Hong Kong (n=19) [25-43] and were published in English. The rest were conducted in mainland China (n=18), stemming from 16 separate research projects [44-61], and half of these articles were published in Chinese [48,53,55-61]. Other than one article examining the incidence of IPV in Hong Kong from 1999 [40], the remaining 36 articles were all published after 2000. Of these, 5 (13.5%) appeared between 2020 and 2022. Most of the publications employed a cross-sectional survey design (n=23, 62.2%), while 12 were longitudinal cohort studies (32.4%). The only randomized controlled trial was conducted in Hong Kong [42]. Nearly all of the studies (n=36, 97.3%) focused on pregnant women, with 15 articles exclusively targeting adult women [25-27,29-36,42,45,56,57]. The sample sizes varied from 110 to 12,044, and 18 studies (48.6%) had a sample size ranging from 1,001 to 12,044 [25,26,28,30-32,34-36,38,44,49,50,57-61]. Participants were primarily recruited from hospitals (n=24, 80%). Data collection was conducted exclusively through face-to-face interviews. The prevalence of IPV was evaluated in 29 studies, of which nearly two-thirds (n=17, 58.6%) utilized the Chinese Abuse Assessment Screen (AAS).

### Research content

#### Intimate partner violence incidence

A total of 29 studies (96.7%) investigated the prevalence of IPV (Table 2). Of these, 20 studies (69.0%) focused on IPV prevalence during pregnancy, with rates ranging from 2.2% [49] to 31.3% [54]. Twelve studies reported a prevalence higher than 10% [25,26,33-36,39,46-48,51,52,54,55,57,60,61]. Thirteen studies provided detailed calculations of the proportions of different types of violence, with psychological violence being the most common (n=11). Among the 12 studies (41.4%) that assessed IPV prevalence over a lifetime, the overall prevalence varied from 5% [56] to 33.7% [25,26]. In 11 studies, the prevalence exceeded 10%, and in six studies, it was greater than 20% [25,26,28,29,37,50,53]. The six studies examining IPV prevalence before pregnancy reported rates between 3.0% [56] and 24.6% [25,26]. In the 10 studies (34.5%) that evaluated IPV in the year preceding the survey, the reported prevalence ranged from 5.0% [42] to 20.7% [27]. Seven studies (24.1%) investigated IPV from 1 month to 3 years postpartum, with results varying from 0.5% to 33.7% [25,26,33,43,55,59-61]. Two studies explored the patterns of IPV from pre-pregnancy to the postpartum period, finding lower IPV prevalence during pregnancy than before pregnancy (14.3% vs. 24.6% and 4.3% vs. 9.1%) [26,44]. Additionally, one study indicated that nearly 13% of victims experienced continuous IPV from preconception through the postnatal period [26].

#### Intimate partner violence-related variables

Twenty studies (66.7%) investigated IPV-related variables using an ecological model [24]. IPV-related factors at the individual level (age, Hong Kong origin or Hong Kong residence status, educational level, health risk behaviors, personal health problems, and adverse childhood experiences), relationship level (marital status, couple relationship, family relationship, pregnancy planning, and social support), community level (employment status and economic status), and societal level (perception on IPV) are presented in Table 3.

Frequently identified risk factors related to IPV during pregnancy included poor family economic conditions (n=9, 45.0%) unintended pregnancy (n=9, 45.0%) partners having health risk behaviors (drinking, smoking, gambling, and drug use) (n=8, 40.0%) poor employment status of women or partners (n=6, 30.0%), low educational levels in women or their partners (n=6, 30.0%), physical or mental health problems in women (n=5, 25.0%), and poor couple relationships (n=4, 20.0%). Three studies each (15.0%) also identified age younger than 25 years for women, adverse childhood experiences, in-law conflicts, and incorrect perceptions of IPV as risk factors. Good social support during pregnancy (n=4, 20.0%) [25,26,33,48,53] and first-time pregnancy were identified as protective factors against IPV during pregnancy.

#### Key outcomes of the studies

Eighteen studies (60.0%) investigated the adverse impact of IPV on maternal health and offspring (Table 4). These adverse effects can be divided into four categories: maternal health, obstetric complications, parenting issues, and the impact on children. Beyond direct physical injury, the maternal effects of IPV during pregnancy primarily manifest as mental health issues, including stress, anxiety, depression, insomnia, and even suicidal tendencies [32,38,39,45,47,49,51,58-60]. Obstetric complications linked to IPV include vaginal bleeding, preterm birth, and placental abruption, among others [51,54,59,60]. Some women may even opt for pregnancy termination [37]. Parenting challenges associated with IPV include difficulties with breastfeeding and the neglect and abuse of children [27,35,55]. The negative impact on children can lead to sleep problems [58] and contribute to issues with mood, emotions, and behavior [46,55]. Additionally, a study found that women who experienced IPV during pregnancy had newborns with shorter telomere lengths in their umbilical cord blood cells than those without such exposure [29].

Five studies (16.7%) investigated perpetrators and found that the perpetrators of IPV in China were mainly husbands and boyfriends [36,37,39,40,44]. Nine studies (30%) investigated female victims’ responses and found that 3.2% to 23.8% were fearful of the perpetrators [25,32,39,52], and some women would fight back or retaliate against the perpetrators [44,50]. Women who experienced IPV in China rarely sought help from professionals such as social workers, medical workers, and the police. Instead, most of them primarily turned to family members or friends for assistance [37,40,44,56]. A small proportion did not seek help from anyone. Further details can be found in Supplementary Material 2.

## Discussion

Our analysis of research on IPV during pregnancy in China revealed that researchers in Hong Kong have devoted more attention to this issue, while research in mainland China remains at a relatively nascent stage. There is an established research team at the University of Hong Kong. Since the publication of the first article on the prevalence of IPV during pregnancy in Hong Kong in 1999 [40], subsequent studies have investigated the prevalence and nature of violence [26,31,34,36,37], explored risk factors associated with IPV during pregnancy [26,28,30,31,33,34], examined its adverse consequences [25,27,29,32,35,36,38,39,41], and sought solutions [42]. Since the dynamics of IPV in Chinese society may differ from those in Western societies, Hong Kong researchers have adapted the AAS, a commonly used violence measurement tool, to reflect conditions in China and have tested its reliability [43]. In contrast, research within mainland China has been geographically limited to a few areas, such as Hunan, Guangdong, and Tianjin City. Furthermore, while most articles by researchers in Hong Kong were published in English in well-known international journals, approximately half of the articles from mainland China were published in Chinese in domestic journals [48,53,55-61]. Additionally, some studies from mainland China [44,50,54,56-61] utilized researcher-designed questionnaires without providing information on the reliability and validity of their measurements, casting doubt on the survey results. Consequently, there is a need for more research employing standardized measurements to accurately represent the status of IPV during pregnancy in mainland China.

### Participants

The research participants were mainly women, who are usually victims of IPV. Survey reports may be underreported due to concerns among victims about losing face and being subjected to ridicule. Meanwhile, perpetrators may conceal their abusive behavior towards female partners to avoid punishment. To address this issue, a study in Hong Kong surveyed male and female partners separately to assess the incidence of IPV. The study found that 84% of the reports were consistent [28]. Concurrently investigating both perpetrators and victims can also yield insights into the motivations and causes of violence, thus providing evidence to address the problem. Considering that other parties relevant to IPV in China include family members such as parents-in-law or parents, neighbors, and government agencies like residents’ committees, women’s federations, and district police stations, it is important to include these stakeholders in future research.

Relatively few studies have focused on disadvantaged groups. Nearly half of the studies (n=15, 40.5%) focused on adults [25-27,29-36,42,45,56,57], potentially limiting our understanding of the situation among pregnant adolescents. In mainland China, there is a notable absence of research specifically targeting underdeveloped rural areas, impoverished Western regions, and areas populated by ethnic minorities. However, multiple studies have indicated that socially disadvantaged individuals are more susceptible to IPV. For instance, a survey revealed that 28.83% of women in rural China have experienced long-term IPV [11], and it has been reported that disabled women in these areas are particularly vulnerable to trafficking and forced childbirth [62]. China, as a multi-ethnic nation, presents unique challenges. Ethnic minorities, who generally have lower levels of education and tend to marry and have children at younger ages compared to the Han population [63], are at increased risk. Additionally, a low educational level has been consistently identified as a strong predictor of abuse during pregnancy [64], suggesting that these individuals may be more vulnerable to IPV. Therefore, efforts to address IPV during pregnancy should pay special attention to the poor, disabled, ethnic minorities, and those experiencing adolescent or unmarried pregnancies. Since these subgroups may be more likely to seek care at smaller clinics, future research should prioritize these populations and incorporate a variety of recruitment sites to ensure a more comprehensive understanding.

### Survey method

In China, research on IPV during pregnancy has mainly utilized traditional face-to-face survey methods. However, given the cultural emphasis on saving face among Chinese people, incidents of IPV, often viewed as family scandals, are seldom disclosed to outsiders [11]. Previous research indicates that a significant number of victims remain silent after experiencing abuse from their partners [37,40,44,50,56]. Therefore, face-to-face surveys may not fully capture the prevalence of IPV. In contrast, the anonymity of online platforms may encourage individuals to speak more freely. According to a report by the Chinese government, as of June 2023, internet usage in China has reached 1.079 billion people, representing a 76.4% penetration rate [65]. Many young women use the internet to access information on maternal and child healthcare [66,67]. There are thousands of apps in China specifically designed for maternal and child healthcare [68], which also provide a space for women to discuss personal experiences. For instance, a search for “domestic violence during pregnancy” on Meiyou (美柚; https://www.meiyou.com/), a popular app in mainland China that primarily assists women in tracking their menstrual cycles [69], yields numerous posts and responses about IPV during pregnancy. The data from online application platforms may offer new insights into the broader scope of IPV in China.

### Research content

The studies in this review that further investigated the reactions of female IPV victims highlighted that Chinese women are relatively uninclined to actively seek assistance. For instance, research conducted in Hong Kong revealed that fewer than 2% of victims were willing to disclose IPV to social workers or their obstetricians [40]. A significant challenge in addressing IPV in China may be the victims’ reluctance to seek help or their tendency to avoid stating the truth. A contributing factor could be the inadequate awareness among Chinese women about IPV. A survey in Shanghai found a high rate of incorrect responses to questions such as whether rape can occur between married couples [56]. The situation may be even more dire in rural areas. Alternatively, the reluctance to seek help could stem from concerns such as the fear of losing face or being stigmatized, financial dependence on partners, concern over partners being penalized for IPV, or the potential for causing family financial problems. Future research should focus more on understanding the internal processes and obstacles faced by individuals experiencing IPV.

#### Intimate partner violence prevalence

The prevalence of IPV during pregnancy in China varied substantially across studies. In most research, the prevalence of psychological violence exceeded that of physical violence, which in turn was more common than sexual violence. This variation may reflect the actual differences in prevalence rates across different regions of China. For instance, the lowest reported rate of IPV during pregnancy was in Shanghai, China’s most developed city, at 2.2% [54], while the highest was in Anhui, a relatively less economically developed province, at 31.3% [56]. However, these results could also be attributed to methodological differences among the studies, such as the use of self-designed questionnaires. The AAS is a reliable and commonly used tool to investigate IPV [69, 70], but it lacks a clear definition of psychological violence in its English version. This can lead to significant discrepancies in results due to varying interpretations of psychological violence. To address this issue and facilitate the use of this instrument in China, researchers in Hong Kong have translated the AAS into Chinese and the Chinese AAS provided explicit definitions of psychological violence. This includes “cold violence,” which involves the withdrawal of verbal and physical communication, and economic violence—both of which are widely recognized within the Chinese context of psychological violence [43]. The Chinese AAS has proven to be satisfactorily accurate in measuring IPV among Chinese women [43]. Most studies in Hong Kong utilized the Chinese AAS to determine the prevalence of IPV during pregnancy, with reported rates ranging from 4.3% to 14.3%. These figures are relatively high compared to those in other developed countries [71]. Future research should focus on the use of standardized measurement tools to enable consistent comparisons across different regions within China and internationally.

#### Intimate partner violence-related variables

In addition to personal factors (e.g., young age and low education level), relationship factors (e.g., poor relationships with partners or family members), community factors (e.g., poor employment status), and social factors (e.g., societal acceptance of IPV), a significant variable related to IPV during pregnancy in China was found to be whether the pregnancy was intended [25,30,34,39,40,48,54,59,60]. Unintended pregnancies, particularly those occurring outside of marriage, may lead to mental or financial stress, potentially escalating conflicts. However, the specific reasons for this association require further investigation. Other factors associated with IPV during pregnancy are the presence of physical or mental illnesses in women, such as chronic diseases and depression [25,28,30,33,61], as well as a lack of social support during pregnancy [25,33,48,53]. One study found that IPV was less likely to occur during a woman’s first pregnancy [30]. Over the past several decades, mainland China’s strict one-child policy has limited the birth of multiple children. However, with the recent promotion of the two-child and three-child policies, the issue of IPV during pregnancy may become more pronounced, warranting additional research.

In addition, the recent emergence of high bride prices in mainland China may contribute to the occurrence of IPV. Bride prices are the payments in money or goods that the groom’s family makes to the bride’s family [72], a tradition deeply rooted in Chinese culture [73]. The recent surge in bride prices can be attributed to the skewed ratio of marriageable men to women [74]. These high bride prices, along with other wedding-related costs, can impose significant financial strain on many families [74]. It remains unclear whether this financial burden may lead men to take out their frustrations on their brides, potentially sparking family conflicts.

#### Adverse outcomes of intimate partner violence

In addition to affecting the mother, IPV during pregnancy can also have detrimental effects on the health of the offspring. For instance, a Hong Kong study found shortened telomeres in the umbilical cord blood of newborns [29]. However, these findings are not consistent with research conducted in other regions, indicating a need for further investigation to determine if there are ethnic or regional disparities [75]. Research from mainland China had found an association between IPV during pregnancy and impairments in the temperament and development of offspring [46,55], a conclusion supported by the outcomes of other similar studies [76,77]. Nonetheless, considering the numerous confounding factors, the impact of prenatal IPV on the temperament and development of offspring necessitates more rigorously designed studies to establish a causal relationship.

#### Interventions

We identified only one study (conducted in Hong Kong) that examined an intervention for addressing IPV during pregnancy. This intervention was grounded in an empowerment framework and administered by healthcare workers [42]. The results indicated that the empowerment-based intervention effectively reduced psychological violence and improved the physical and mental health outcomes of participants [42]. Although this is a single study, it suggests the potential for adapting concepts and methodologies from Western countries to tackle IPV within Chinese society. It also highlights the pivotal role healthcare workers can play in addressing IPV during pregnancy, given their increased contact with IPV victims compared to other professionals. Most of the studies reviewed here recruited participants from medical institutions, demonstrating the practicality of IPV screening in Chinese medical settings. In Hong Kong, further research is necessary to determine whether medical institutions can reliably implement interventions to mitigate IPV. Conversely, in mainland China, there is a need for foundational research on both violence screening and intervention strategies. According to the Anti-Domestic Violence Law of the People’s Republic of China, medical institutions in mainland China are mandated to document the diagnosis and treatment of IPV survivors [78]. Consequently, medical professionals are not legally obligated to perform routine screenings or interventions for IPV. Additionally, studies from mainland China indicate a low level of awareness about IPV among medical workers, with their knowledge on the subject being notably insufficient [56,79]. Many hold the view that IPV is a private issue and are resistant to the idea of medical staff engaging in IPV prevention [56]. Therefore, future research in mainland China should prioritize increasing the overall societal understanding of IPV, particularly among medical professionals, and advocate for the advancement of relevant legislation.

A limitation of this review was that not all databases in China were systematically searched; therefore, some studies published in Chinese may have been inadvertently missed. Additionally, a systematic quality appraisal of the selected studies was not conducted.

However, as the first study to systematically analyze IPV during pregnancy in China, this review establishes a foundation that enhances our understanding of the issue. It also highlights the limitations of the selected studies and offers recommendations for future research. This study plays a crucial role in raising public awareness about IPV in China.

This review systematically summarized the characteristics of 37 studies on IPV during pregnancy in China. There is a need for more research from mainland China, and the use of standardized measurements is essential for robust research design and comparison with other studies. Research in mainland China has been primarily concentrated in a few provinces and has often been investigated from the perspective of victims. Surveys were predominantly conducted in a face-to-face format. The incidence, related variables, and negative impacts of IPV during pregnancy are the primary focus of these studies. Future research should examine multiple facets of this issue, with a particular emphasis on socially disadvantaged groups. The scope of research should also expand to include efforts to increase public awareness of IPV and to explore interventions to address the problem.

IPV during pregnancy is a complex social issue in China, with relevant factors including personal, familial, political, economic, social norms, and other dimensions. This indicates that a multidisciplinary professional collaboration is necessary to address this problem effectively.

## Figures and Tables

**Table 1.** General characteristics of the selected studies (N=37)

Variable	Categories	n (%)	References
Location	Hong Kong	19 (51.4)	[25-43]
	Mainland China	18 (48.6)	[44-61]
Publication year	1991–2000	1 (2.7)	[40]
	2001–2010	19 (51.4)	[30-32,34-39,41-44,50,56,58-61]
	2011–2020	12 (32.4)	[27-29,45-48,51,52,54,55,57]
	2021–2022	5 (13.5)	[25,26,33,49,53]
Publication language	English	28 (75.7)	[25-54]
	Chinese	9 (24.3)	[48,53,55-61]
Participants	Pregnant women	36 (97.3)	[25-27,29-61]
	Male partners	1 (2.7)	[28]
Age of participants (year)	≥16	2 (5.4)	[28,52]
	≥18	15 (40.5)	[25-27,29-36,42,45,56,57]
	Not specified	11 (29.7)	[37,39,40,43,46-51,53]
	NA	9 (24.3)	[38,41,44,54,55,58-61]
Sample size (n)	100–500	8 (21.6)	[27,33,42,43,46,48,55,56]
	501–1,000	11 (29.7)	[29,37,39-41,45,47,51-54]
	1,001–12,044	18 (48.6)	[25-26,28,30-32,34-36,38,44,49,50,57-61]
Sample size calculation	Yes	9 (24.3)	[34-37,39,42,49,52,56]
	No	28 (75.7)	[25-33,38,40,41,43-48,50,51,53,54,55,57,58,59-61]
Study design	Cross-sectional study	23 (62.2)	[26,28,30,31,33-37,40,43,44,49,50,52-54,56-61]
	Cohort study	12 (32.4)	[25,27,29,32,38-39,41,45-47,51,55]
	Case-control study	1 (2.7)	[48]
	RCT	1 (2.7)	[42]
Research setting (n=30)	Hospital	24 (80.0)	[25-27,29-42,45-51,53,54,56,57,59-61]
	Community	4 (13.3)	[28,44,52,58]
	Hospital+community	2 (6.7)	[43,55]
IPV measurement (n=29)	Chinese AAS	17 (58.6)	[25-27,29-40,42,46-49,51-53,55]
	SDQ	7 (24.1)	[44,50,56-57,59-61]
	CTS2	2 (6.9)	[28,45]
	Others	2 (6.9)	[54,58]
	NA	1 (3.4)	[41]

AAS, Abuse Assessment Screen; CTS2, Revised Conflict Tactics Scale; IPV, intimate partner violence; NA, not available; RCT, randomized controlled trial; SDQ, self-designed questionnaire.

**Table 2.** IPV prevalence reported in selected studies (N=29)

Time period	Categories	Prevalence (%)	References
Lifetime (n=12, 41.4% )		33.7 (unidentifiable)	[25,26]
		27.7 (physical: 17.9, sexual: 13.6)	[28]
		23.5 (psychological: 23.3, physical: 3.5, sexual: 1.8). One type: 18.9, two types: 4.3, three types: 0.4	[29]
		27.3 (emotional: 9.8, physical: 8.6, physical+emotional: 5.3)	[37]
		11.5 (unidentifiable)	[38]
		17.1 (unidentifiable)	[39]
		17.9 (unidentifiable)	[40]
		12.6 (emotional: 5.6, physical: 3.5, sexual: 8.0)	[44]
		22.6 (physical: 7.8, emotional: 3.0, sexual: 18.1)	[50]
		22.2 (unidentifiable)	[53]
		5.0 (men 5.0)	[56]
		10.5 (unidentifiable)	[58]
Pre-pregnancy (n=6, 20.7%)		24.6 (unidentifiable)	[25,26]
		22.9 (unidentifiable)	[33]
		9.1 (emotional: 1.9, physical: 3.9, sexual: 5.8)	[44]
		21.7 (emotional: 2.8, physical: 7.5, sexual: 17.3)	[50]
		13.3 (mental 10.8, physical: 3.5, sexual: 1.2)	[53]
		3.0 (mental)	[56]
Preceding year (n=10, 34.5%)		20.7 (unidentifiable)	[27]
		18.8 (physical: 11.9, sexual: 9.1)	[28]
		9.1 (emotional: 6.7, physical + sexual : 2.5)	[30-32]
		Psychological: 8.8, physical: 4.1, sexual: 5.5, injury 2.0. One type: 5.3%, two types: 3.5%, three types: 1.5%, four types: 0.9%	[34-36]
		Physical: 6.1, sexual: 9.8	[37]
		16.6 (sexual: 1.7)	[39]
		15.7 (threats: 4.1, sexual: 9.4)	[40]
		15.9 (unidentifiable)	[41]
		5.0 (unidentifiable)	[42]
		11.07 (psychological: 9.23, physical: 4.55, sexual: 2.34)	[45]
During pregnancy (n=20, 69.0%)	Less than 10% (n=8, 27.6%)	6.5 (unidentifiable)	[30-32]
		4.3 (unidentifiable) (1–3 times: 14.8, ≥5 times: 7.4)	[40]
		4.3 (emotional: 1.5, physical: 1.1, sexual: 2.8)	[44]
		2.2 (mental 1.6; physical: 0.6, sexual: 0.2)	[49]
		7.0 (emotional: 1.3, physical: 1.8, sexual: 4.9)	[50]
		8.8 (mental 8.2; physical: 0.6, sexual: 0.0)	[53]
		2.5 (mental)	[56]
		6.4 (psychological: 4.5, physical: 1.5, sexual: 0.0)	[59]
	Higher than 10% (n=12, 41.4%)	14.3 (psychological: 14.1, physical: 0.9, sexual: 0.4)	[25,26]
		13.5 (unidentifiable)	[33]
		11.2 (unidentifiable)	[34-36]
		10.4 (all were verbal/sexual)	[39]
		11.3 (psychological: 7.0, psychological+sexual: 3.9, psychological+physical: 0.5)	[46-48]
		18.32 (psychological: 14.3, physical: 2.1, sexual: 0.3)	[51]
		15.62 (mental 11.07; physical: 0.98, sexual: 0.86). Two types: 3.08	[52]
		31.3 (psychological: 28.8, physical: 7.0, sexual: 2.3)	[54]
		20.4 (unidentifiable)	[55]
		11.57 (psychological: 5.87, physical: 3.57, sexual: 2.14)	[57]
		16.8 (unidentifiable)	[60]
		11.5 (unidentifiable)	[61]
Post-delivery (n=7, 24.1%)	1 month	14.3 (unidentifiable)	[25,26]
		14.7 (unidentifiable)	[33]
		0.5 (mental)	[55]
	1 year	8.3 (emotional: 2.5, physical: 3.2, sexual: 4.9)	[44]
		17.4 (unidentifiable)	[59]
		33.7 (unidentifiable)	[60]
		26.1 (unidentifiable)	[61]
	3 years	11.8 (unidentifiable)	[33]

The terms “emotional,” “physical,” “psychological,” and “sexual” refer to the corresponding types of violence.

**Table 3.** IPV-related variables (N=20)

Category	IPV predictor	n (%)	References
Individual level	Age	5 (25.0)	[34,43,54,58,59]
	Hong Kong origin or Hong Kong residence status	2 (10.0)	[34,40]
	Educational level	6 (30.0)	[30,44,54,57,58,61]
	Health risk behavior of woman or partner (smoking, drinking, drug use)	7 (35.0)	[28,30,44,48,54,57,59,60]
	Personal health problems (depression, chronic disease, etc.)	5 (25.0)	[25,28,30,33,61]
	Adverse childhood experiences (witnessing violence, etc.)	3 (15.0)	[33,44,48]
	Marital status (long marriage, unfree love marriage)	3 (15.0)	[34,44,59]
Relationship level	Poor couple relationship	4 (20.0)	[34,44,50,54]
	Family relationships (living with extended family, conflict with other family members, etc.)	3 (15.0)	[28,30,52]
	Pregnancy-related factors (unintended pregnancy, first pregnancy)	9 (45.0)	[25,30,34,39,40,48,54,59,60]
	Social support (partner support, support from other family members)	4 (20.0)	[25,33,48,53]
Community level	Employment status of partner or woman	6 (30.0)	[34,37,40,44,57,61]
	Economic status (income, indebtedness, needing security assistance or not, living conditions)	10 (50.0)	[30,33,34,37,43,48,52,57,58,61]
Societal level	Incorrect perceptions of IPV	3 (15.0)	[48,59,60]

IPV, Intimate partner violence.

**Table 4.** Key findings of the studies that reported outcomes (N=17)

Category	Outcome	n (%)	Reference
Maternal	Stress, anxiety or depression	8 (47.1)	[32,39,45,47,49,51,59,60]
	Poor mental health-related QoL	3 (17.6)	[32,38,58]
	Self-harm thinking	1 (5.9)	[32]
	Poor physical health-related QoL	2 (11.8)	[36,38]
	Skin injury	1 (5.9)	[59]
	Poor sleeping, insomnia	3 (17.6)	[58-60]
	Poor life confidence	1 (5.9)	[58]
Obstetric	Pregnancy termination	1 (5.9)	37]
	Adverse birth outcomes (vaginal bleeding, neonatal asphyxia, PTB, LBW, placental abruption, stillbirth)	4 (23.5)	[51,54,59,60]
Children	Reduced newborn telomere length	1 (5.9)	[29]
	Sleeping problems	1 (5.9)	[58]
	Children’s temperament and development (had higher scores in distractibility on the RITQ, mood, emotions)	2 (11.8)	[46,55]
Parenting	Women were less likely to initiate breastfeeding	2 (10.5)	[35,55]
	Less consistency in parenting	1 (5.3)	[55]
	Physical child abuse	1 (5.3)	[27]

LBW, Low birth weight; PTB, preterm birth; QoL, quality of life; RITQ, Revised Infant Temperament Questionnaire.

## Supplementary materials

Further details on supplementary materials are presented online (available at https://doi.org/10.4069/whn.2024.03.16).
