# Supplementary material for: The status of intimate partner violence against pregnant women in contemporary China: a scoping review
Source: Womens Health Nurs. 2024 Mar 29;30(1):41–55. doi: 10.4069/whn.2024.03.16 (PMC11073557; doi:10.4069/whn.2024.03.16)
Supplement: Supplementary material 1. [file whn-2024-03-16-Supplementary-material-1.pdf]

WHN

Supplementary Material 1. Article search process in the Cochrane Library

ID	Search	Hits
#1	MeSH descriptor: [Intimate Partner Violence] explode all trees	577
#2	MeSH descriptor: [Domestic Violence] explode all trees	1,132
#3	MeSH descriptor: [Spouse Abuse] explode all trees	225
#4	MeSH descriptor: [Gender-Based Violence] explode all trees	16
#5	MeSH descriptor: [Battered Women] explode all trees	75
#6	MeSH descriptor: [Stalking] explode all trees	4
#7	Intimate partner violence or domestic violence or domestic abuse* or family violence or spous * abuse* or dat* violence or gender*based violence or violence against women or batter* female* or batter* women or marital violence or marital abuse* or stalking or partner abuse* or maternal abuse*	12,173
#8	#1 or #2 or #3 or #4 or #5 or #6 or #7	12,691
#9	MeSH descriptor: [Pregnancy] explode all trees	31,435
#10	MeSH descriptor: [Pregnant Women] explode all trees	774
#11	Pregnant women or expectant mother* or pregnancy	82,117
#12	#9 or #10 or #11	82,570
#13	MeSH descriptor: [East Asian People] explode all trees	200
#14	MeSH descriptor: [China] explode all trees	8,370
#15	MeSH descriptor: [Hong Kong] explode all trees	965
#16	MeSH descriptor: [Macau] explode all trees	6
#17	Chinese or China or Hong Kong or HK or Macau or Macao	123,377
#18	#13 or #14 or #15 or #16 or #17	123,472
#19	#8 and #12 and #18	129
