# Supplementary material for: The status of intimate partner violence against pregnant women in contemporary China: a scoping review
Source: Womens Health Nurs. 2024 Mar 29;30(1):41–55. doi: 10.4069/whn.2024.03.16 (PMC11073557; doi:10.4069/whn.2024.03.16)
Supplement: Supplementary material 2. [file whn-2024-03-16-Supplementary-material-2.pdf]

Supplementary Table 2. Key information of selected studies (N=30)

Study No.	First author [reference]	Publication year	Publication language	General characteristics of the selected studies								Key outcomes of the study	
				Research aim	Study design	IPV measure instrument	Participants	Participants' age (yr, mean ± SD) and marital status n (%)	Research setting	IPV prevalence (%)	IPV-related variables	IPV-related adverse health outcome	Other results
R1	Chan [25]	2021	English	To examine the effect of father involvement and IPV on the association between unintended pregnancy and postnatal depression	One prospective cohort study	C-AAS	1,083 Adult pregnant women	Age: ≥ 18, 31.3 ± 4.6<br>Married, 982 (90.7); unmarried, 91 (8.5)	In 4 public hospitals in Hong Kong	During pregnancy: 14.3	Women with unintended pregnancies more likely to experience IPV (p < .01), fear result from IPV (p < .01)		Fear of perpetrator: 3.5%
R1	Chan [26]	2022	English	To investigate the trajectory of IPV before, during, and after pregnancy<br>To examine the predictive factors of these IPV-related categories	One longitudinal cross-sectional survey	C-AAS	1,083 Adult pregnant women	Age: ≥ 18, 31.3 ± 4.6<br>Married, 982 (90.7); unmarried, 91 (8.5)	In 4 public hospitals in Hong Kong	Lifetime: 33.7<br>Pre-pregnancy: 24.6<br>During pregnancy: 14.3<br>Postdelivery: 14.3	Risk factors: stress (aOR, 1.09) and depression (aOR, 1.07) during pregnancy<br>Protective factor: partner emotional support (aOR, 0.66) and perceived good social support (aOR, 0.98) during pregnancy		
R2	Chan [27]	2012	English	To investigate the impact of pregnancy violence on child maltreatment	One longitudinal cohort study	C-AAS	487 Pregnant women (184 abused, 303 non-abused in the 2005 study)	Age: ≥ 18, 34.4 ± 4.8<br>Married/cohabited: 469 (96.3); divorced/separated, 18 (3.7)	In 7 public hospitals in Hong Kong	Preceding year: 20.7		IPV during pregnancy was associated with lifetime (aOR, 1.74) and preceding year (aOR, 1.78) child physical maltreatment	
R3	Can [28]	2011	English	To discuss if pregnancy is a risk factor for IPV	One cross-sectional study	C-CTS2	2,225 Male partners	Age: ≥ 16, 50 ± 13.2<br>Married, 2,181 (98.0); cohabiting, 44 (2.0)	Communities in Hong Kong	Lifetime: 27.7 (phy, 17.9; sex, 13.6)<br>Preceding year: 18.8 (phy, 11.9; sex, 9.1)	Risk factors: male partner was drug abused, alcohol abused, has depression and suicide ideation, had poor anger management, and experienced in-law conflicts		
R4	Chan [29]	2019	English	To examine the association between IPV against women before childbirth and cord blood telomere length in their newborn	One longitudinal cohort study	C-AAS	774 Adult pregnant women	Age: ≥ 18, 31.05 ± 4.5<br>Married, 698 (92.1); single/divorced, 60 (7.9)	In 1 public hospital in Hong Kong	Lifetime: 23.5 (phy, 3.5; psy, 23.3; sex, 1.8)<br>One type, 18.9; 2 types, 4.3; 3 types, 0.4		Psychological abuse and sexual abuse were significantly associated with the shorter newborns' telomere length	

(Continued on the next page)

Supplementary Table 2. Continued

Study No.	First author [reference]	Publication year	Publication language	General characteristics of the selected studies								Key outcomes of the study	
				Research aim	Study design	IPV measure instrument	Participants	Participants' age (yr, mean ± SD) and marital status n (%)	Research setting	IPV prevalence (%)	IPV-related variables	IPV-related adverse health outcome	Other results
R5	Chan [30]	2009	English	To quantify in-law conflict level and to examine correlates of in-law conflict and violence against pregnant women	One cross-sectional study	C-AAS	3,245 Chinese pregnant women	Age: ≥ 18 , 30.8 ± 4.9<br>Married/cohabiting, 3,156 (97.3); single/divorced/separated, 87 (2.7)	In 7 hospitals in Hong Kong	Preceding year: 9.1 (emo, 6.7; phy or sex, 2.5)	Risk factors: in-law conflict, chronic illness, dependence on social support, unplanned pregnancy, number of children, receipt of social security, indebtedness, alcohol, were strongly associated with preceding year abuse<br>Protective factor: women were well-educated, first-time pregnancy		
R5	Tiwari[31]	2007	English	To report the prevalence and type of IPV victimization and examine socioeconomic influences on IPV	One cross-sectional study	C-AAS	3,245 Chinese pregnant women	Age: ≥ 18 , 30.8 ± 4.9<br>Married/cohabiting, 3,156 (97.3); single/divorced/separated, 87 (2.7)	In 7 hospitals in Hong Kong	Preceding year: 9.1 (psy, 6.7; phy and/or sex, 2.5; psy and phy/sex, 1.4)<br>During pregnancy: 6.5			
R5	Tiwari [32]	2008	English	To assess the impact of psychological IPV on mental health	One longitudinal cohort study	C-AAS	3,245 Chinese pregnant women	Age: ≥ 18, 30.8 ± 4.9<br>Married/cohabiting, 3,156 (97.3); single/divorced/separated, 87 (2.7)	In 7 hospitals in Hong Kong	Last year: 9.1 (psy, 6.7; phy and/or sex, 2.5; both psy and phy/sex, 1.4)		Psychological abuse increase the risk of postnatal depression (aOR, 1.84), self-harming thinking (aOR, 3.50), psychological and physical abuse lead to poor mental-related quality of life	Fear of perpetrator: psy abuse, 12.6%; phy or sex, 23.8%

(Continued on the next page)

https://doi.org/10.4069/whn.2024.03.16

Supplementary Table 2. Continued

Study No.	First author [reference]	Publication year	Publication language	General characteristics of the selected studies						IPV prevalence (%)	IPV-related variables	Key outcomes of the study	
				Research aim	Study design	IPV measure instrument	Participants	Participants' age (yr, mean ± SD) and marital status n (%)	Research setting			IPV-related adverse health outcome	Other results
R6	Chen [33]	2022	English	To identify categories of IPV from pre-pregnancy to 3 years after childbirth<br>To investigate risk and protective factors associated with the different categories	One longitudinal cross-sectional study	C-AAS	340 Adult pregnant women to attend their first-time prenatal care	Age: ≥ 18, 31.30 ± 4.26<br>Married, 315 (92.6); divorced/separated, 25 (7.4)	In 1 hospital in Hong Kong	Before pregnancy: 22.9<br>During pregnancy: 13.5<br>4-week PD: 14.7<br>3-yr PD: 11.8<br>Violent relationship, 11.8; decreased violent relationship, 20.6; nonviolent relationship, 67.6	Risk factors: receiving social security assistance (aOR, 6.44), adverse childhood experiences (aOR, 1.25), perinatal depression (aOR, 1.06), stress (aOR, 1.12), anxiety (aOR, 1.29)<br>Protective factor: father involvement, family support, better mental health in HRQoL (aOR, 0.96)		
R7	Lau [34]	2005	English	To identify the prevalence and nature of violence in Hong Kong<br>To explore the risk factors associated with abuse during pregnancy	One retrospective cross-sectional study	C-AAS/ C-CTS2	1,200 Chinese postnatal mothers	Age: 18–47/ abused, 28.67 ± 6.58; non-abused, 20.30 ± 5.08<br>Married, 1,134 (94.5); single/ cohabiting, 66 (5.5)	In 1 hospital in Hong Kong	During pregnancy: 11.2<br>Preceding year: phy, 4.1; psy, 8.8; sex, 5.5; injury, 2.0<br>Two types, 11.2–33.6; 3 types, 8.2–16.4; 4 types, 8.2	Risk factors: women younger than 25, born in Hong Kong, was single or cohabiting, poor partner relationship, partner was underemployment, need financial support exclusively by partner, small size of residence, and an unplanned pregnancy		
R7	Lau [35]	2007	English	To examine IPV and postnatal depression as potential correlates of breastfeeding	One correlational cross-sectional study	C-AAS/ C-CTS2	1,200 Chinese postnatal mothers	Age: 18–47/ abused, 28.67 ± 6.58; non-abused, 20.30 ± 5.08<br>Married, 1,134 (94.5); single/ cohabiting, 66 (5.5)	In 1 hospital in Hong Kong	During pregnancy: 11.2 (AAS)<br>Preceding year: phy, 4.1; psy, 8.8; sex, 5.5; injury, 2.0		No IPV during pregnancy (aOR, 1.84) more likely to initiate breastfeeding	
R7	Lau [36]	2008	English	To understand the types of IPV during pregnancy and to examine its effect on HRQoL	One retrospective cross-sectional study	C-AAS/ C-CTS2	1,200 Chinese postnatal mothers	Age: 18–47/ abused, 28.67 ± 6.58; non-abused, 20.30 ± 5.08<br>Married, 1,134 (94.5); single/ cohabiting, 66 (5.5)	In 1 hospital in Hong Kong	During pregnancy: 11.2<br>Preceding year: phy, 4.1; psy, 8.8; sex, 5.5; injury, 2.0<br>One type, 5.3; 2 types, 3.5; 3 types, 1.5; 4 types, 0.9<br>More than one type, 5.9		Women who had experienced different types of IPV were associated with lower scores in the majority of domains of the SF-36	Perpetrator: husband, 81.3%; boyfriends, 8.0%; ex-husband, 0.7%; ex-boyfriends, 11.3%

(Continued on the next page)

Supplementary Table 2. Continued

Study No.	First author [reference]	Publication year	Publication language	General characteristics of the selected studies: Research aim	Study design	IPV measure instrument	Participants	Participants' age (yr, mean ± SD) and marital status n (%)	Research setting	IPV prevalence (%)	IPV-related variables	Key outcomes of the study: IPV-related adverse health outcome	Other results
R8	Leung [37]	2002	English	(1) To compare the prevalence of DV among abortion seeking patients with other general gynecology patients; (2) to see if a follow-up interview (6 weeks after abortion) can improve the abuse disclosure rate; (3) to see if the abused victims accept a direct referral to their gynecologist/social workers for help	One prospective cross-sectional study	C-AAS	501 Patients (245 seeking abortion patients, 256 general gynecology patients)	Age: pregnancy termination, 27.0 ± 8.0, non-pregnancy termination, 37.7 ± 12.1<br>Married, 307 (61.28); unmarried, 192 (38.32)	In 1 hospital in Hong Kong	Lifetime (phy, emo, sex): 27.3 (phy or emo, 23.7; emo only, 9.8; phy only, 8.6; both emo and phy, 5.3)<br>Preceding year: phy, 6.1; sex, 9.8	Risk factors: have a low monthly family income, partner is a manual worker or unemployed	The abortion seeking group had a significantly higher prevalence of abuse and tended to suffer more severe physical abuse<br>27.3% of victims' decision for termination of pregnancy had been affected by their abusive experience	Abortion seeking group:<br>Physical abuse perpetrator: boyfriends, 33.3%; husbands, 13.3%<br>Sexual abuse perpetrator: boyfriends and husbands were equal (45.8% vs. 50.0%)<br>Help-seeking: 18.8% of participants with recent abuse would like to reveal abuse to their gynecologists and/or social workers
R9	Leung [38]	2005	English	To evaluate the impact of IPV on the baseline quality of life among different groups of patients	One retrospective cohort study	C-AAS	1,614 Obstetric or gynecological patients (abortion-seeking group, 300; infertility patients, 500; general gynecology patients, 300; obstetric antenatal care patients, 514)	Age: NA<br>Married, 1,350 (83.6); single/cohabiting, 264 (16.4)	In 1 hospital in Hong Kong	Lifetime: 7.2 (abortion-seeking group and obstetric antenatal care patients, 11.5)		The mean QoL scores among the abused victims were significantly lower in the physical health, social relationship, environment, and psychological health domains	
R10	Leung [39]	2002	English	To study the relationship between DV and postnatal blues/depression in a Chinese community	One prospective cohort study	C-AAS	838 Postdelivery women	Age: abused, 29.2 ± 5.4; non-abused, 29.9 ± 5.0<br>Married, 815 (97.3); single/divorced, 23 (2.7)	In 1 hospital in Hong Kong	Lifetime: 17.1<br>Last year: 16.6 (sex, 1.7)<br>Current pregnancy: 10.4<br>All abuse were verbal and/or sexual	Risk factors: pregnancy was more likely to be unplanned in the abused group	The abused group had significantly higher SDSS and EPDS scores	Perpetrator: husband/boyfriends, 27.9%; mother-in-law, 26.7%; employer/colleague, 20.9%<br>Fear perpetrator: 3.2%

(Continued on the next page)

https://doi.org/10.4069/whn.2024.03.16

Supplementary Table 2. Continued

Study No.	First author [reference]	Publication year	Publication language	General characteristics of the selected studies						IPV prevalence (%)	IPV-related variables	Key outcomes of the study	
				Research aim	Study design	IPV measure instrument	Participants	Participants' age (yr, mean ± SD) and marital status n (%)	Research setting			IPV-related adverse health outcome	Other results
R11	Leung [40]	1999	English	To study the incidence of DV	One cross-sectional study	C-AAS	631 First-time antenatal care pregnant women	Age: abused, 29.1 ± 5.5, non-abused, 29.6 ± 5.2. Married, 589 (93.3); single/divorced, 42 (6.7)	In 1 hospital in Hong Kong	Lifetime: 17.9<br>Last year: 15.7 (sex, 9.4; threats of abuse, 4.1)<br>Current pregnancy: 4.3 (1–3 times, 14.8; ≥ 5 times, 7.4)	Risk factors: being ethnically Chinese, was Hong Kong permanent resident, unplanned pregnancy, having partners who were unemployed or manual workers		Perpetrator: husband (86.9% of all victims, 77.8% during pregnancy, 96.6% sex)<br>Help-seeking: 2% of victims want to reveal to medical social worker; no victim want to reveal to obstetricians
R12	Leung [41]	2001	English	To see whether the pregnancy outcome would be affected even if the nature of violence is not physical injury	One cohort study	NA	584 Pregnant women	Age: NA<br>Marital status: NA	In 1 Hospital in Hong Kong	Preceding years: 15.9		No obstetric differences between the abused and non-abused group	
R13	Tiwari [42]	2005	English	To evaluate the effectiveness of an empowerment intervention in reducing<br>IPV and improving health status	One RCT	C-AAS/CTS form research	110 Adult Chinese pregnant women	Age: ≥ 18/experimental, 30.0 ± 5.1, control, 30.1 ± 5.2<br>Married, 100 (90.9); single/divorced, 10 (9.0)	In 1 hospital in Hong Kong	Last year: 5			Experimental group had higher physical functioning, improved role limitation, less psychological abuse, less minor physical violence, lower prenatal depression scores, and more bodily pain
R14	Tiwari [43]	2007	English	To assess the measurement accuracy and the utility of the C-AAS	One cross-sectional study	C-AAS/CTS2	257 Chinese women (100 pregnant and 157 non-pregnant)	Age: 36.2 ± 8.1<br>Married, 235 (91.4); unmarried, 22 (8.6)	In 1 hospital and a community center in Hong Kong		Age difference between the couple and the woman's need for financial assistance was associated with IPV		Specificity of C-AAS, > 89%; sensitivity, 36.3%–65.8%; positive predictive values, > 80%; negative predictive values, 66%–93%

(Continued on the next page)

Supplementary Table 2. Continued

Study No.	First author [reference]	Publication year	Publication language	General characteristics of the selected studies						IPV prevalence (%)	IPV-related variables	Key outcomes of the study	
				Research aim	Study design	IPV measure instrument	Participants	Participants' age (yr, mean ± SD) and marital status n (%)	Research setting			IPV-related adverse health outcome	Other results
R15	Guo [44]	2004	English	To examine patterns of physical and sexual abuse before, during, and after pregnancy	One cross-sectional study	SDQ	12,044 Mothers with 6 to 18-month children	Age: NA<br>Marital status: NA	In 32 communities in North China (Henan, Liaoning, Shanxi, Tianjin province)	Lifetime: 12.6 (emo, 5.6; sex, 8.0; phy 3.5)<br>Pre-pregnancy (mean, 12 months): 9.1 (emo, 1.9; sex, 5.8; phy, 3.9)<br>During pregnancy: 4.3 (emo, 1.5; sex, 2.8; phy, 1.1)<br>After delivery (mean, 11 months): 8.3 (emo, 2.5; sex, 4.9; phy, 3.2)<br>Abuse by period: before pregnancy only, 3.2; during pregnancy only, 0.6; after delivery only, 2.5, before and during pregnancy 0.5, before and after pregnancy 2.5, during and after pregnancy 0.5, in all three periods 2.8.<br>Types of abuse: emo only, 10.5; sex only, 41.7; phy only, 19.8; emo+sex, 3.6; emo+phy, 6.2; phy+sex, 10.5; emo+phy+sex, 7.7<br>Frequency:1–2 times, 36.6; ≥ 3 times, 63.4<br>Phy severity: minor, 80; severe, 20	Risk factors: woman has a low-level education, was alcohol abuser, was violence witness, poor couple relationship, married for a long time, living in extended family or living alone, husband was blue-collar worker, has low income, or drug abuse, abuse happened before pregnancy<br>Risk factors of postpartum abuse: abuse before and during pregnancy		Perpetrators: husbands<br>Reaction to physical abuse: hit back, 44.6%; never hit back, 11.2%<br>Help-seeking: don't tell anyone, 34.0%; tell family member, 32.4%; tell friends, 24.9%
R16	Hou [45]	2020	English	To explore the contribution of IPV to perinatal depression	One longitudinal cohort study	C-CTS2S	813 Pregnant women in 13 gestational weeks	Age: ≥ 18, 20–46, 30.78 ± 4.39<br>Marital status: NA	In 2 hospitals in Changsha City and Yiyang City, Hunan province, Mainland China	Last year: 11.07 (phy, 4.55; psy, 9.23; sex, 2.34)		Victims had higher EPDS scores than non-victims at all time points<br>The decreasing slope for victims' postnatal depression trajectory was slower than non-victims	

(Continued on the next page)

https://doi.org/10.4069/whn.2024.03.16

Supplementary Table 2. Continued

Study No.	First author [reference]	Publication year	Publication language	General characteristics of the selected studies						IPV prevalence (%)	IPV-related variables	Key outcomes of the study	
				Research aim	Study design	IPV measure instrument	Participants	Participants' age (yr, mean ± SD) and marital status n (%)	Research setting			IPV-related adverse health outcome	Other results
R17	Zou [46]	2015	English	To investigate the effects of DV on their infant's development in China	One cohort study	C-AAS	223 Mothers (DV 86, non-violence 137) and infants	Age: abused, 27.76 ± 2.74; non-abused, 27.24 ± 2.96<br>Marital status: NA	In 1 hospital in Hunan province, Mainland China	During pregnancy: 11.3 (psy, 7.0; psy+sex, 3.9; psy+phy, 0.5)		Child of victims had higher Vein Glu, GABA, cortisol level and higher score in the distractibility of the RITQ<br>The Vein Glu and GABA were positively correlated with infants' mood	
R17	Zhang [47]	2012	English	To investigate the prevalence of DV and postnatal depression, and to explore the relationship between DV and postnatal depression	One prospective cohort study	C-AAS	846 Postdelivery women	Age: abused, 28.0 ± 3.0; non-abused, 28.0 ± 3.0<br>Marital status: NA	In 1 hospital in Hunan province, Mainland China	During pregnancy: 11.3 (psy, 7.0; psy+sex, 3.9; psy+phy, 0.5)		Psychologic violence during pregnancy was a risk factor for postnatal depression (aOR, 4.032)	
R17	Zhang [48]	2013	Chinese	To explore the relationship between some psychological factors and DV during pregnancy	One case-control study	C-AAS	86 Abuse pregnant women and 137 non-abused pregnant women	Age: abused, 27.76 ± 2.74; non-abused, 27.24 ± 2.96<br>Marital status: NA	In 1 hospital in Hunan province, Mainland China	During pregnancy: 11.3 (psy, 7.0; psy+sex, 3.9; psy+phy, 0.5)	Risk factors: husband smoking, high family income, woman's acceptance of violence, witness to violence, unexpected pregnancy abortion history, poor social support		
R18	Wu [49]	2022	English	To establish the prevalence of IPV among pregnant women in Shenzhen during the COVID-19 pandemic<br>To explore the association between IPV and prenatal anxiety and depression	One cross-sectional study	C-AAS	3,434 Pregnant women	Age: 28.97 ± 4.57<br>Married, 3,188 (92.83); unmarried/ divorced/ widowed, 246 (7.16)	In hospitals from 10 administrative areas in Shenzhen, Mainland China	During pregnancy: 2.2 (mental, 1.6, phy, 0.6; sex, 0.2)		IPV victims were more likely to develop prenatal anxiety (OR, 4.207) and prenatal depression (OR, 3.864)	

(Continued on the next page)

Supplementary Table 2. Continued

Study No.	First author [reference]	Publication year	Publication language	General characteristics of the selected studies						IPV prevalence (%)	IPV-related variables	Key outcomes of the study	
				Research aim	Study design	IPV measure instrument	Participants	Participants' age (yr, mean ± SD) and marital status n (%)	Research setting			IPV-related adverse health outcome	Other results
R19	Wu [50]	2005	English	To investigate the prevalence, type, and severity of DV, and determine the factors related to DV	One cross-sectional study	SDQ	1,215 Abortion seeking women	Age: 27.4 ± 5.6<br>Married, 932 (76.7); single, 277 (22.8)	In 8 hospitals in North China (Tianjin, Liaoning, Henan, Shaanxi province)	Lifetime: 22.6 (phy, 7.8; emo, 3.0; sex, 18.1)<br>Before current pregnancy: 21.7 (phy, 7.5; emo, 2.8; sex, 17.3)<br>During current pregnancy: 7.0 (phy, 1.8; emo, 1.3; sex, 4.9)<br>After pregnancy: 0.8<br>Experienced continuous violence: 28.4<br>One type, 77; 2 types, 16.1; 3 types, 4.4	Risk factors: many times induced abortion, have bad relationship with partner		Reaction to physical violence: never retaliated, 15%; retaliated every time, 23.1%<br>Help-seeking: not telling anyone, 49.4%; tell friends, 27.2%; tell family members, 26.1%
R20	Yu [51]	2018	English	To examine the associations among IPV during pregnancy, prenatal depression, and adverse birth outcomes	One cohort study	C-AAS	797 Third-trimester pregnant women	Age: 27.4 ± 4.2<br>Marital status: NA	In 3 hospitals in Hubei province, Mainland China	During pregnancy: 18.32 (psy, 14.3; phy, 2.1; economic, 2.0; sexual, 0.3)		IPV was significantly associated with prenatal depression and adverse birth outcomes (neonatal asphyxia, PTB, and LBW)	
R21	Zheng [52]	2020	English	To explore the prevalence and association between family factors and DV	One cross-sectional survey	C-AAS	813 Third-trimester adult pregnant women	Age: ≥ 16, 28.98 ± 4.52<br>Married, 728 (89.5)	In 14 urban communities of Hengyang City, Hunan province, Mainland China	During pregnancy: 15.62 (phy, 0.98; men, 11.07; sex, 0.86)<br>Two types, 3.08	Risk factors: tensions with mother-in-law and other family members (OR, 2.85), medium household debt (OR, 2.17), family dysfunction (OR, 2.01)		Fear perpetrator: 5.41%
R22	Zeng [53]	2021	Chinese	To investigate the IPV prevalence, and to explore the IPV correlates	One cross-sectional survey	C-AAS	510 Postpartum women	Age: < 35, 455 (89.2%); ≥ 35, 55 (10.8%)<br>Marital status: NA	In 1 hospital in Hunan province, Mainland China	Lifetime: 22.2<br>Pre-pregnancy: 13.3 (phy, 3.5; men, 10.8; sex, 1.2)<br>During pregnancy: 8.8. (phy, 0.6; men, 8.2; sex, 0.0)	Adverse pregnancy history was the risk factor for IPV before pregnancy (OR, 2.941)<br>Subjective social support was the protective factor of pregnancy during pregnancy (OR, 0.803)		

(Continued on the next page)

https://doi.org/10.4069/whn.2024.03.16

Supplementary Table 2. Continued

Study No.	First author [reference]	Publication year	Publication language	General characteristics of the selected studies								Key outcomes of the study	
				Research aim	Study design	IPV measure instrument	Participants	Participants' age (yr, mean ± SD) and marital status n (%)	Research setting	IPV prevalence (%)	IPV-related variables	IPV-related adverse health outcome	Other results
R23	Zhu [54]	2016	English	To estimate the IPV during pregnancy in 2 cities of Anhui; to investigate the association of spontaneous PTB with IPV during pregnancy	One cross-sectional survey	Question list generated by WHO.	928 Third-trimester pregnant women	Age: NA<br>Marital status: NA	In 2 hospitals in Anhui province, Mainland China	During pregnancy: 31.3 (phy, 7.0; psy, 28.8; sex, 2.3)	Risk factors: be younger (women, < 25; partner, < 30), woman less educated, unwanted pregnancy, inadequate communication, partner drinking or smoking before pregnancy	Psychological violence plus physical or sexual violence was significantly associated with spontaneous PTB (aOR, 2. 31)	
R24	Zhong [55]	2013	Chinese	To investigate the effects of DV on the temperament and behavior of preschool offspring	One cohort study	C-AAS	196 Mothers with preschool children	Age: NA<br>Marital status: NA	In hospitals and communities	During pregnancy: 20.4		Lower breastfeeding rate, lower consistency of parenting<br>Children have a higher score for affective symptoms and overall difficulty, lower score for the nature of emotion	
R25	Huang [56]	2008	Chinese	To understand the current situation of maternal DV in Shanghai	Cross-sectional study	SDQ	200 Postpartum women	Age: 19–38, 25.96 ± 3.27<br>Married, 199 (99.5); single, 1 (0.5)	In 1 hospital in Shanghai, Mainland China	Lifetime: 5.0<br>Before pregnancy: 3.0<br>During pregnancy: 2.5<br>After pregnancy: 1.0.<br>All were mental violence			Cognition on DV: poorly understood in 3 questions: "does rape exist in couples," "can the life value achieve when the women cannot be pregnant," and "is the husband cursing wife a kind of DV."<br>Help-seeking: 1) seeking help from parents, 2) calling the police, 3) temporarily leaving partner
R26	Liu [57]	2016	Chinese	To investigate the prevalence and influential factors of pregnancy DV in Shenzhen	Cross-sectional study	SDQ	7,820 Postpartum women	Age: 19–43, 28.92 ± 6.78<br>Marital status: NA	In 8 hospitals in Shenzhen, Guangdong Province, Mainland China	Perinatal IPV: 11.57 (psy, 5.87; phys, 3.57; sex, 2.14)	Risk factors: low family income, partner has low-level education, partner was alcoholic, women was a manual worker		

(Continued on the next page)

Supplementary Table 2. Continued

Study No.	First author [reference]	Publication year	Publication language	General characteristics of the selected studies						IPV prevalence (%)	IPV-related variables	Key outcomes of the study	
				Research aim	Study design	IPV measure instrument	Participants	Participants' age (yr, mean ± SD) and marital status n (%)	Research setting			IPV-related adverse health outcome	Other results
R27	Liu [58]	2003	Chinese	To investigate the incidence rate of IPV and its influence on psychosomatic health of mothers and infants	Cross-sectional study	The questionnaire of DV on pregnant women compiled by Women and Children Health Center of Pecking University	3,025 Mothers of infants aged 6–18 months	Age: NA<br>Marital status: NA	In 8 districts of Tianjin City, Mainland China	Any time: 10.5 (one type, 8.2; ≥ two types, 2.2)	Risk factors: women younger than 25, was less educated, has low income	The abused women had more poor sleeping, bad emotions, and poor life confidence<br>Infants in abused group had more sleeping problem, bad emotion, more aggressive	
R28	Ye [59]	2005	Chinese	To investigate the incidence of DV during pregnancy and postpartum and its health hazards to victims	Cross-sectional study	SDQ	1,438 Women waiting for delivery or attending postpartum follow-up	Age: NA<br>Marital status: NA	In 4 hospitals in Guangzhou and Shenzhen Cities, Guangdong province, Mainland China	During pregnancy: 6.4 (psy, 4.5; phy, 1.5; sex, 0; psy+phy, 0.4)<br>42 days postpartum: 17.4	Risk factors: pregnant women older than 35, unfree love marriage, unintended pregnancy, woman has incorrect perception of IPV, partner has drinking, gambling behavior	Stress, anxiety, insomnia, depressed skin injury, vaginal bleeding, and PTB	
R29	Ye [60]	2005	Chinese	To investigate the current situation of DV among pregnant and postpartum women, and to analyze its causes and Influencing factors	Cross-sectional study	SDQ	1,397 Women waiting for delivery or attending postpartum follow-up	Age: NA<br>Marital status: NA	In 5 hospitals in Henan province, Mainland China	During pregnancy: 16.8<br>Postpartum: 33.7	Risk factors: unintended pregnancy, partner has risk behavior (drinking, smoking, gambling, drug abuse...), woman has incorrect perception of IPV	Stress, anxiety, insomnia, depressed<br>Physical injury (skin injury, fracture)<br>Adverse obstetric outcomes (vaginal bleeding, stillbirth, placental abruption, PTB)	
R30	Fan [61]	2006	Chinese	To investigate the current status of DV among women during pregnancy and postpartum in different section in China; to analyze the reasons and possible factors which will lead to DV	Cross-sectional study	SDQ	2,835 Pregnant or postpartum women	Age: NA<br>Marital status: NA	In hospitals in Henan and Guangdong provinces, China	During pregnancy: 11.5 (Henan, 16.8; Guangdong, 6.4)<br>During postpartum: 26.1 (Henan, 33.7; Guangdong, 17.4)	Risk factors: low-level education, low family income, low-income occupation, poor living status		

aOR, Adjusted odds ratio; C-AAS, Chinese version of the Abuse Assessment Screen; CTS2, the Revised Conflict Tactics Scale; CTS2S, Short Form of the Revised Conflict Tactics Scale; DV, domestic violence; EPDS, Edinburgh Postnatal Depression Scale; Glu, glutamate; GABA, gamma-aminobutyric acid; HRQoL, health related quality of life; IPV, intimate partner violence; LBW, low birth weight; NA, not available; psy, psychological violence; phy, physical violence; PTB, preterm birth; RITQ, Revised Infant Temperament Questionnaire; SDQ, self-designed questionnaire; sex, sexual violence; SDSS: Stein Daily Scoring System.

https://doi.org/10.4069/whn.2024.03.16
